# Reviewed article: Brasil JAI, Raposo LM. Occupational patterns and risks of occupational accidents with biological material: A cross-sectional study, Brazil, 2015-2019. Epidemiol Serv Saude. 2025:34;e20250051

**DOI:** 10.1590/S2237-96222025v34e20250051.b

**Published:** 2025-10-27

**Authors:** Jônathas de Lima Arruda

**Affiliations:** 1 Universidade Federal de Pernambuco, Recife, PE, Brazil

## Peer review B

## Questionnaire

Does the manuscript contain new and significant information to justify publication? Yes

Does the Abstract (Summary) clearly and accurately describe the content of the article? Yes

Is the problem significant and concisely stated? Yes

Are the methods described comprehensively? Yes

Are the interpretations and conclusions justified by the results? Yes

Is adequate reference made to other work in the field? Yes

## Manuscript Structure

Length of article is: Adequate

Number of tables is: Adequate

Number of figures is: Adequate

Please state any conflict(s) of interest that you have in relation to the review of this paper. None

Interest: Excellent

Quality: Excellent

Originality: Excellent

Overall: Excellent

Recommendation: Minor Revision

## Comments

INTRODUÇÃO

2º parágrafo - Os impactos psicológicos e sociais da infecção pelo HIV são distintos das demais IST, dado o histórico de profunda discriminação e estigma às pessoas que vivem com HIV. No trecho “Os principais agentes patogênicos envolvidos em acidentes de trabalho envolvendo material biológico incluem os vírus das hepatites B e C e o vírus da imunodeficiência humana, cujas consequências podem ser devastadoras para a saúde física, social e psicológica dos trabalhadores afetados”, os autores se referem somente ao HIV ou incluem as hepatites no trecho? Não ficou claro.

DISCUSSÃO

4º parágrafo - No trecho “Apesar do maior conhecimento técnico e da escolaridade superior, a presença de vínculos laborais informais e a predominância masculina nesse grupo contribuem para a negligência nos protocolos de segurança” os autores afirmam que pertencer ao gênero masculino é um fator de fragilidade para acidentes ocupacionais. Por quê? Há mais literatura que faça a correlação entre o gênero masculino e maiores taxas de acidentes ocupacionais? É interessante um novo trechopara aprofundar especificamente essa discussão?

Os autores trouxeram um excelente texto e com dados preciossos sobre acidentes ocupacionais no país. Acredito ser oportuno trazer um parágrafo que aborde a falta de dados sobre adesão à profilaxia pós-exposição para o HIV após acidentes laborais. Existe um vácuo analítico que pode ser explorado.

Também cabe uma discussão sobre a maior quantidade de pessoas não brancas e menos escolarizadas no grupo 1; grupo esse que também possui uma cobertura vacinal deficiente.

